# Vitamin C and folate status in hereditary fructose intolerance

**DOI:** 10.1038/s41430-022-01178-3

**Published:** 2022-07-19

**Authors:** Ainara Cano, Carlos Alcalde, Amaya Belanger-Quintana, Elvira Cañedo-Villarroya, Leticia Ceberio, Silvia Chumillas-Calzada, Patricia Correcher, María Luz Couce, Dolores García-Arenas, Igor Gómez, Tomás Hernández, Elsa Izquierdo-García, Dámaris Martínez Chicano, Montserrat Morales, Consuelo Pedrón-Giner, Estrella Petrina Jáuregui, Luis Peña-Quintana, Paula Sánchez-Pintos, Juliana Serrano-Nieto, María Unceta Suarez, Isidro Vitoria Miñana, Javier de las Heras

**Affiliations:** 1 Biocruces Bizkaia Health Research Institute, 48093 Barakaldo, Spain; 2 Food Research, AZTI, Basque Research and Technology Alliance (BRTA), Parque Tecnológico de Bizkaia, Astondo Bidea, Edificio 609, 48160 Derio, Spain; 3 Paediatrics Unit, Río Hortega University Hospital, 47012 Valladolid, Spain; 4 Metabolic Diseases Unit, Department of Paediatrics, Ramón y Cajal Hospital, 28034 Madrid, Spain; 5 Department of Metabolism Diseases and Nutrition, Niño Jesús University Children´s Hospital, 28009 Madrid, Spain; 6 Internal Medicine Service, Cruces University Hospital, 48903 Barakaldo, Spain; 7 12 de Octubre University Hospital, CIBERER, MetabERN, 28041 Madrid, Spain; 8 Nutrition and Metabolic diseases Unit, La Fe University Hospital, 46026 Valencia, Spain; 9 Unit of Diagnosis and Treatment of Congenital Metabolic Diseases, Department of Pediatrics, IDIS-Health Research Institute of Santiago de Compostela. CIBERER. MetabERN. Santiago de Compostela University Clinical Hospital, 15704 Santiago de Compostela, Spain; 10 Department of Paediatric Gastroenterology, Hepatology and Nutrition, Sant Joan de Déu Hospital, 08950 Barcelona, Spain; 11 Araba University Hospital, 01009 Vitoria-Gasteiz, Spain; 12 Paediatric Service, Albacete University Hospital, 02006 Castilla-La Mancha, Spain; 13 Pharmacy Department, Infanta Leonor University Hospital, 28031 Madrid, Spain; 14 Clinical Nutrition Section, Navarra University Hospital, 31008 Pamplona, Spain; 15 Paediatric Gastroenterology, Hepatology and Nutrition Unit, Mother and Child Insular University Hospital complex, Asociación Canaria para la Investigación Pediátrica (ACIP), CIBEROBN. University Institute for Research in Biomedical and Health Sciences, University of Las Palmas de Gran Canaria, 35016 Las Palmas de Gran Canaria, Spain; 16 Paediatric Service, Málaga Regional University Hospital (HRU), 29010 Málaga, Spain; 17 Biochemistry Laboratory, Metabolism Area, Cruces University Hospital, 48903 Barakaldo, Spain; 18 Division of Paediatric Metabolism, CIBERER, MetabERN, Cruces University Hospital, 48093 Barakaldo, Spain; 19 Department of Paediatrics, University of the Basque Country (UPV/EHU), 48940 Leioa, Spain

**Keywords:** Metabolic disorders, Nutrition

## Abstract

**Background:**

Hereditary fructose intolerance (HFI) is a rare inborn error of fructose metabolism caused by the deficiency of aldolase B. Since treatment consists of a fructose-, sucrose- and sorbitol-restrictive diet for life, patients are at risk of presenting vitamin deficiencies. Although there is no published data on the status of these vitamins in HFI patients, supplementation with vitamin C and folic acid is common. Therefore, the aim of this study was to assess vitamin C and folate status and supplementation practices in a nationwide cohort of HFI patients.

**Methods:**

Vitamin C and folic acid dietary intake, supplementation and circulating levels were assessed in 32 HFI patients and 32 age- and sex-matched healthy controls.

**Results:**

Most of the HFI participants presented vitamin C (96.7%) and folate (90%) dietary intake below the recommended population reference intake. Up to 69% received vitamin C and 50% folic acid supplementation. Among HFI patients, 15.6% presented vitamin C and 3.1% folate deficiency. The amount of vitamin C supplementation and plasma levels correlated positively (*R* = 0.443; *p* = 0.011). Interestingly, a higher percentage of non-supplemented HFI patients were vitamin C deficient when compared to healthy controls (30% vs. 3.1%; *p* = 0.036).

**Conclusions:**

Our results provide evidence for the first time supporting vitamin C supplementation in HFI. There is great heterogeneity in vitamin supplementation practices and, despite follow-up at specialised centres, vitamin C deficiency is common. Further research is warranted to establish optimal doses of vitamin C and the need for folic acid supplementation in HFI.

## Introduction

Hereditary fructose intolerance (HFI; OMIM 229600) is a rare, autosomal-recessive inborn error of metabolism caused by deficiency of the enzyme fructose-1,6-bisphosphate aldolase (aldolase B; E.C. 4.1.2.13) [1]. Aldolase B regulates both glycolysis and gluconeogenesis, catalysing various reactions such as cleavage of fructose-1-phosphate and reversible cleavage of fructose-1,6-bisphosphate. It is expressed in the cytoplasm of hepatocytes, renal tubules, and in the small intestine [2]. Although the prevalence of HFI is estimated to be between 1:20,000 and 1:31,000 in certain European populations, recent data shows that HFI may have a wider distribution and higher prevalence than expected (circa 1:10,000) [3].

Symptoms in HFI patients usually initiate 5 or 6 months after birth due to the introduction of complementary feeding in the infant. After fructose ingestion, children usually present failure to thrive, accompanied by nausea, vomiting, and abdominal pain. Persistent intake of fructose may lead to hypoglycaemia, jaundice, metabolic acidosis, seizures, coma, liver cirrhosis, and eventual death [4–6]. Despite HFI treatment, which consists of a fructose-, sucrose- and sorbitol (FSS)-restricted diet for life [7], some HFI patients present long-term complications, such as hepatic steatosis [8].

Vitamin C, also known as L-ascorbic acid, is a water-soluble essential micronutrient naturally found in fresh fruit and vegetables, e.g. oranges, lemons, limes, potatoes, grapefruit, broccoli, red peppers, spinach and tomatoes [9]. This vitamin is involved in various enzyme reactions, such as redox balance maintenance and iron absorption [10]. It has been reported that vitamin C reduces systemic inflammation, helps correct sepsis-induced coagulopathy and prevents vascular injury, and its circulating levels are also inversely associated with metabolic syndrome [11, 12].

Folate is a generic term that typically refers to a group of compounds that belong to the family of B- vitamins. Also known as vitamin B9, folic acid is the synthetic form of folate. This vitamin can be found in legumes, meat, milk, eggs and vegetables like spinach, broccoli, and lettuce [9]. Folate participates in tissue growth, helps in the production of DNA and RNA, contributes to the maturation of erythrocytes and, together with vitamin C, participates in protein metabolism [13]. A deficiency of folate usually results from chronic alcoholism, malabsorption disorders, haemolytic anaemia, increased requirements during pregnancy, or a diet poor in this compound [9].

Since HFI treatment consists of a FSS-restrictive diet for life, HFI patients are at risk of presenting vitamin deficiencies. Although there are no clinical guidelines for this rare disease, supplementation with vitamin C and/or folic acid is common. To the best of our knowledge, there is no published data on vitamin status in HFI patients. Therefore, the aim of this study was to assess vitamin C and folate status and supplementation practices in a nationwide cohort of HFI patients.

## Patients and methods

### Study design and participants

Recruitment for this cross-sectional study was conducted from October 2019 to November 2020. Study visits were carried out at Cruces University Hospital, Spain. The study population for this study comprised 32 genetically diagnosed HFI patients and 32 age- and sex-matched healthy controls. No sample size calculation was performed, all available HFI patients with data on vitamin C and folate status were included in the study.

Eleven Spanish hospitals participated in the study: Cruces University Hospital [host] (*n* = 6), Araba University Hospital (*n* = 2), Albacete University Hospital (*n* = 1), Navarra University Hospital (*n* = 2), 12 de Octubre University Hospital (*n* = 4), Niño Jesús University Children’s Hospital (*n* = 3), Ramón y Cajal University Hospital (*n* = 5), La Fe University Hospital (*n* = 2), Málaga Regional University Hospital (*n* = 1), Río Hortega University Hospital (*n* = 2) and Mother and Child Insular University Hospital (*n* = 4).

All the HFI patients were on a FSS-restricted diet for at least 2 years.

### Ethics

The study protocol was carried out according to the ethical guidelines of the revised 1975 Declaration of Helsinki [14] and approved by the Basque Ethics Committee for Research (CEIm-E), ethical approval code: PI2019072. Written informed consents were obtained from parents or legal guardians of children (below 18 years of age) and adult study participants.

### Dietary intake

Dietary information was collected in a self-administered nutritional record of dietary intake on 3 days (two during the week and one at the weekend) in 30 HFI patients and in 28 non-HFI healthy controls. Once completed, vegetable fibre, vitamin C and folate daily intake were calculated using the Nutritional Calculation DIAL programme (Version 3.10.5.0) [15]. In addition, information regarding vitamin C and folic acid supplementation was collected from all the study participants.

### Biochemical analyses

Circulating concentrations of ascorbate (vitamin C) and folate were determined in all study subjects by routine clinical techniques. Briefly, vitamin C in plasma was quantified by high performance liquid chromatography with electrochemical detection. Vitamin C levels ≤23 μmol/L were considered to be hypovitaminosis and values ≤11 μmol/L as vitamin C deficiency [16, 17]. The quantitative determination of folate in serum was carried out by the Atellica^®^ IM Folate assay for in vitro diagnostic use. Concentrations under 6.8 nmol/L were considered to be folate deficiency [18].

### Statistical analysis

Data are presented as mean ± standard deviation or median and range (minimum-maximum), depending on data distribution. Continuous variables were compared by unpaired Student’s *t* test or Mann–Whitney U test. Differences in categorical variables were assessed using *χ*^2^ or Fisher’s exact test. *P* values were based on two-tailed comparisons and those under 0.05 were considered to indicate a statistically significant difference. In order to assess bivariate relationships between variables, Pearson or Spearman Rank test correlation analyses were used. All the statistical analysis tests were subject to data distribution and were carried out using SPSS software, version 23 for Windows (IBM, Chicago, IL).

## Results

### Study cohort

The study population comprised 32 genetically diagnosed HFI patients and 32 healthy controls, all of them of Caucasian origin. Clinical characteristics of the study participants are shown in Table 1. There were no significant differences in weight, body mass index (BMI), age, and sex between HFI and control subjects (Table 1).

**Table 1:** Clinical, biochemical, and nutritional characteristics in HFI patients and healthy controls.

	Healthy controls	HFI patients	*p*value
*n*	32	32	
Male/female, *n*/*n*	13/19	12/20	0.798
Age, years	16.0 [2.1–61.3]	14.6 [5.5–63.5]	0.961
Weight, kg	50.5 ± 15.3	47.2 ± 15.6	0.401
BMI, kg/m^2^	20.2 ± 3.2	19.0 ± 3.1	0.710
Plasma vitamin C (µmol/L)	45.1 [10.2–129.5]	49.4 [5.7–138.0]	0.895
Serum folate (nmol/L)	21.5 [12.7–48.7]	24.7 [6.1–54.4]	0.619
Vitamin C hypovitaminosis/deficiency (*n*; %)	2; 6.3%	7; 21.9%	0.148
Vitamin C deficiency (*n*; %)	1; 3.1%	5; 15.6%	0.196
Folate deficiency (*n*; %)	0	1; 3.1%	1.000

Dietary intake			
*n*	28	30	
Vegetable fiber (g/day)	15.9 [9.4–59.7]	12.1 [6.1–21.4]	**0.006**
Vitamin C (mg/day)	107.0 [42.8–346.6]	23.8 [6.4–76.1]	**<0.001**
Folate (µg/day)	202.2 [115.8–524.2]	183.5 [78.6–304.3]	**0.027**

Continuous variables are represented as mean ± standard deviation or as median [minimum-maximum], depending on data distribution. Significant *p* values are marked in bold. Body mass index (BMI). Vitamin C hypovitaminosis/deficiency: circulating vitamin C levels ≤23 µmol/L. Vitamin C deficiency: circulating vitamin C levels ≤11 µmol/L.

### Dietary intake in HFI patients

Vitamin C dietary intake was markedly lower in HFI patients when compared to their controls (23.8 [6.4–76.1] mg/day vs. 107.0 [42.8–346.6]; *p* < 0.001) (Table 1). Folate daily dietary intake was also significantly lower in HFI patients than in controls (183.5 [78.6–304.3] µg/day vs. 202.2 [115.8–524.2] µg/day; *p* = 0.027) (Table 1). Among the HFI participants, 96.7% had a vitamin C dietary intake below the recommended daily vitamin C population reference intake (PRI) and 90% had a folate dietary intake below the recommended PRI (Table 2).

**Table 2:** Vitamin C and folate dietary intake, supplementation and circulating levels in HFI patients (dietary intake data not available in two subjects).

		Vitamin C					Folic acid/folate				
		Dietary intake	Supplementation (mg/day)			Conc. (µmol/L)	Dietary intake	Supplementation (µg/day)			Conc. (nmol/L)
Patient No.	Mult. name	mg/day	Single	Mult.	Total	Plasma	µg/day	Single	Mult.	Total	Serum
1	Supradyn^®^ EE	**50.9**	250	90	340	82.9	**266**	0	100	100	35.1
2		**46.6**	0	0	0	34.1	**145**	0	0	0	**6.1**
3		**40.0**	0	0	0	58.5	**201**	0	0	0	12.0
4		**29.9**	0	0	0	44.9	**168**	0	0	0	8.4
5		**23.8**	0	0	0	**6.2**	**113**	5000	0	5000	38.1
6		**20.6**	200	0	200	38.0	**130**	2100	0	2100	11.3
7		**12.1**	250	0	250	94.8	**189**	5000	0	5000	54.4
8		**21.7**	250	0	250	73.8	**184**	0	0	0	13.4
9	ViMin 50 NM	**64.1**	0	30	30	42.6	**208**	0	100	100	16.5
10	ViMin 50 NM	**41.5**	0	30	30	19.3	**122**	0	100	100	8.6
11		**35.4**	0	0	0	**5.7**	**257**	0	0	0	11.1
12		**11.5**	20	0	20	76.7	206	0	0	0	42.1
13		**13.3**	500	0	500	39.7	141	2500	0	2500	47.1
14		**12.1**	0	0	0	76.7	**79**	0	0	0	15.2
15	Supradyn^®^ AW	**15.5**	0	80	80	**6.8**	**144**	5000	200	5200	32.0
16	Supradyn^®^ Active	**13.4**	0	80	80	**7.4**	**158**	5000	200	5200	54.4
17		**17.5**	0	0	0	31.8	**216**	0	0	0	17.7
18		**19.3**	0	0	0	26.7	**197**	0	0	0	15.9
19			40	0	40	82.9		0	0	0	24.5
20	Supradyn^®^ Active	**39.2**	0	80	80	103.3	**153**	0	200	200	12.9
21	Supradyn^®^ Prot.	**15.6**	46	20	66	95.4	**132**	700	0	700	45.8
22	ViMin 50 NM		0	30	30	23.1		0	100	100	31.5
23	Nu U nutrition	**16.4**	1000	100	1100	138.0	**129**	0	200	200	38.5
24	Supradyn^®^ Prot.	**76.1**	225	29	254	60.2	**229**	300	0	300	29.5
25		**28.3**	50	0	50	67.0	**172**	300	0	300	38.5
26		**23.8**	200	0	200	39.2	**208**	0	0	0	18.6
27		**22.5**	200	0	200	76.1	**183**	0	0	0	17.4
28		**42.0**	0	0	0	**6.8**	**304**	0	0	0	24.9
29		**6.4**	1000	0	1000	53.9	**132**	0	0	0	31.3
30		76.1	50	0	50	21.6	281	0	0	0	25.8
31		**66.6**	0	0	0	57.3	**304**	0	0	0	10.7
32	Supradyn^®^ Prot.	**43.4**	0	20	20	57.9	**195**	200	0	200	54.4

“Total” column refers to the sum of single vitamin and multivitamin supplementation. Dietary intake below population reference intake (PRI) [16, 22] and vitamin levels indicating deficiency [16–18] are marked in bold. *Mult.* Multivitamin, *EE* Extra Energy, *AW* Active Woman, *Prot.* Protovit.

### Vitamin C and folic acid supplementation

**Fig. 1:**
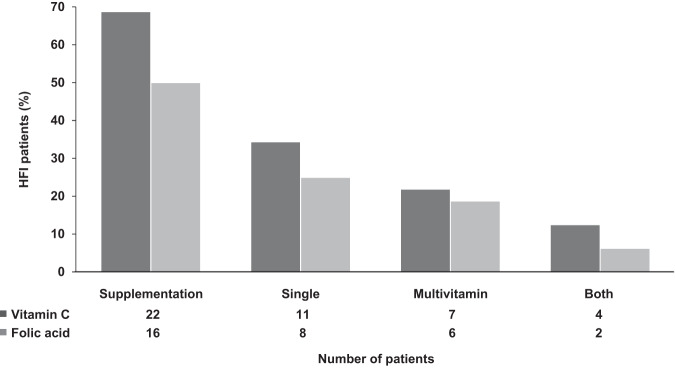
Vitamin C and folic acid supplementation in HFI patients (*n* = 32). The percentage of subjects that received vitamin C and folic acid is represented in dark and light grey columns, respectively.

Table 2 shows vitamin C and folic acid dietary intake, supplementation and circulating levels in HFI participants. Of the 32 HFI patients studied, 22 (69%) received vitamin C and 16 (50%) folic acid supplementation from single and/or multivitamin supplements (Fig. 1). Eleven HFI patients (34%) were prescribed different multivitamin supplements containing, among others, different amounts of vitamin C and folic acid (Table 2). In contrast, no control subject took either vitamin C or folic acid supplements. All the different multivitamin supplements consumed by the study participants and depicted in Table 2 contain both vitamin C and folic acid, except Supradyn^®^ Protovit, which does not contain folic acid.

### Vitamin C status

**Fig. 2:**
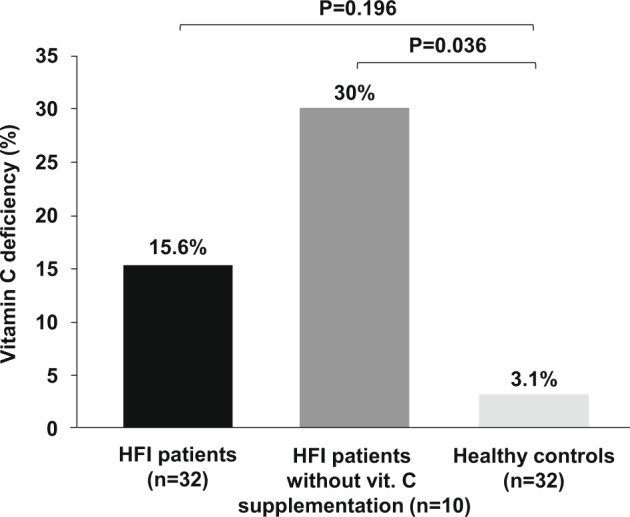
Vitamin C deficiency in HFI patients and healthy controls. Columns represent the percentage of vitamin C deficiency in HFI patients altogether (*n* = 32), HFI patients without vitamin C supplementation (*n* = 10), and healthy controls (*n* = 32).

Among the HFI patients, 7 (21.9%) presented vitamin C hypovitaminosis/deficiency, with 5 patients (15.6%) with circulating vitamin C values below 11 μmol/L. Twenty-two out of 32 HFI patients received vitamin C supplementation, and there were no differences in plasma vitamin C levels or the percentage of vitamin C deficiency between HFI patients and healthy controls (Table 1). Taking into account vitamin C supplementation, although there were no significant differences in vitamin C levels between non-supplemented HFI patients and healthy controls (32.9 [5.7–76.7] μmol/L vs. 45.1 [10.2–129.5] μmol/L; *p* = 0.154), a higher percentage of non-supplemented HFI patients presented vitamin C deficiency (30% vs. 3.1%; *p* = 0.036) (Fig. 2).

**Fig. 3:**
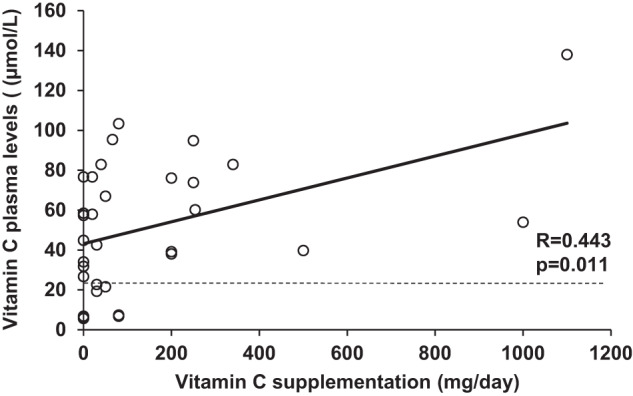
Correlation between the amount of vitamin C supplementation and plasma levels in HFI patients. The horizontal dashed line indicates the lower limit of normal for vitamin C plasma concentrations (23 µmol/L).

Within the HFI group, although the patients that were not given vitamin C supplements presented lower circulating levels than those who were given supplements (32.9 [5.7–76.7] μmol/L vs. 59.1 [6.8–138] μmol/L; *p* = 0.047), there were not statistically significant differences in the percentage of vitamin C deficiency (30% vs. 9.1%; *p* = 0.293). The amount of vitamin C supplementation and plasma levels correlated positively (*R* = 0.443; *p* = 0.011) (Fig. 3).

### Folate status

**Fig. 4:**
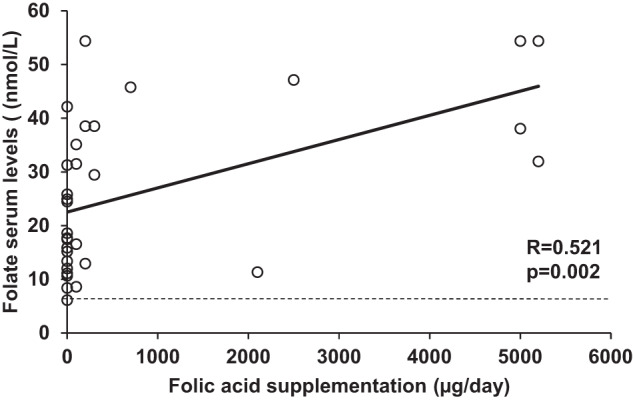
Correlation between the amount of folic acid supplementation and serum folate levels in HFI patients. The horizontal dashed line indicates the lower limit of normal for folate serum concentrations (6.8 nmol/L).

There were no significant differences in folate serum levels between HFI patients and their controls and, among all study participants, only one HFI patient presented folate deficiency (Table 1). HFI patients that did not receive folic acid supplementation presented lower levels of serum folate than healthy controls (16.7 [6.1–42.1] nmol/L vs. 21.5 [12.7–48.7] nmol/L; *p* = 0.017), and also than HFI patients that received supplementation (16.7 [6.1–42.1] nmol/L vs. 36.6 [8.6–54.4] nmol/L; *p* = 0.04). In HFI patients, the amount of folic acid supplementation and circulating folate levels correlated positively (*R* = 0.521; *p* = 0.002) (Fig. 4).

### Multivitamin vs. single supplementation

#### Vitamin C

Seven HFI patients received vitamin C in the form of a multivitamin only, eleven from single vitamin C supplements, and four patients received both (Fig. 1). Without considering the four patients that received vitamin C from both supplement forms, the patients with single vitamin C supplementation received a higher amount of this micronutrient than those with multivitamin supplementation (200 [20–1000] mg/day vs. 30 [20–80] mg/day; *p* = 0.028). There were no statistically significant differences in circulating vitamin C levels (67.0 [21.6–94.8] μmol/L vs. 22.7 [6.8–103.3] μmol/L; *p* = 0.368), or in the percentage of vitamin C deficiency (28.6% vs. 0%; *p* = 0.137) between HFI patients with single and multivitamin supplementation.

#### Folic acid

Eight HFI patients received single folic acid supplements, six were given multivitamin supplements, and two patients received both (Fig. 1). Without considering the two HFI patients that received folic acid from both supplement forms, those patients with single folic acid supplementation received a higher daily amount of this vitamin than those with multivitamin supplementation (1400 [200–5000] µg/day vs. 100 [100–200] µg/day; *p* = 0.001). In accordance, those patients with single folic acid supplementation presented higher serum folate levels than those who received it from multivitamin supplementation (42.1 [11.3–54.4] nmol/L vs. 24.0 [8.6–38.5] nmol/L; *p* = 0.05).

## Discussion

Patients with HFI display an innate aversion to sweet foods, fruit and vegetables [19], and HFI treatment consists precisely of a FSS-restricted diet for life [6]. Therefore, these patients are at risk of presenting some vitamin deficiencies. Since to the best of our knowledge there is no published data on vitamin status in HFI patients, the aim of this study was to assess vitamin C and folate status and supplementation practices in a nationwide cohort of HFI patients.

Our data shows that, although most HFI patients (69%) received vitamin C supplementation, vitamin C hypovitaminosis/deficiency was common. Noticeably, five HFI patients (15.6%) presented vitamin C plasma levels below 11 μmol/L, regarded by some authors as severe deficiency [16, 20]. Concerning folic acid, 50% of HFI patients received supplementation, and only one patient presented folate deficiency.

In this study we have used the PRI values, which indicate the level of nutrient intake that is adequate for virtually all people in a population group [21], suggested by the European Food Safety Authority as the dietary reference values for vitamin C and folic acid [16, 22]. In our cohort of HFI participants, the majority presented vitamin C (96.7%) and folate (90%) daily dietary intake below the recommended PRI. Vitamin C dietary intake was especially low in HFI patients compared to the healthy controls, due to the fact that fruit and vegetables are the main sources of vitamin C. HFI patients under treatment consume scarce amounts of vegetables and, when they eat them, they usually boil them to lower their content in fructose. The fact that vitamin C is a temperature-sensitive vitamin and is easily degraded during boiling [23] makes it is even harder to reach the recommended vitamin C dietary reference value for patients with HFI.

There are no clinical guidelines for HFI management and, although there is no published data on vitamin status in HFI patients except for some anecdotal clinical cases [24, 25], supplementation with vitamin C and/or folic acid is common. The most relevant finding of the present study is that the HFI patients that did not consume vitamin C supplements presented a higher percentage of vitamin C deficiency than the healthy control subjects, providing for the first time evidence for the indication of vitamin C supplementation in patients with HFI under a FSS-restricted diet.

Regarding folate, patients that did not receive folic acid supplementation presented lower circulating levels than healthy controls and supplemented HFI patients. However, there was only one HFI patient with folate deficiency. This observation is reasonable as vitamin C is found only in fresh fruits and vegetables, which are very limited in the diet of HFI patients, whereas folate can be found also in other food sources permitted in their diet, such as meat, milk, and eggs [7].

Our study, with data of HFI patients from 11 different centres, shows great heterogeneity in clinical practice regarding folic acid and vitamin C supplementation in HFI patients in Spain. This can be explained by the lack of clinical guidelines and data on vitamin status in this rare disease. Furthermore, the variability in the nutritional approach that we describe in this study is common in other rare inherited metabolic disorders like phenylketonuria, urea cycle disorders, and methylmalonic acidemia [26–28].

Moreover, in our study, the high percentage of vitamin C hypovitaminosis/deficiency in HFI patients despite supplementation is to be noted, as 18.2 % of HFI patients supplemented with vitamin C presented vitamin C hypovitaminosis/deficiency, and 9.1% vitamin C deficiency. This may be due to the fact that many patients received vitamin C supplementation at a low dosage, mainly those who received it from multivitamin supplementation. Thus, supplementation should be prescribed in order to provide at least the recommended dietary reference values [21], and vitamin status should be monitored regularly.

Once the diagnosis has been made and treatment implemented, HFI is thought to be a benign disease, as when a FSS-restricted diet is started early in life and adherence is maintained the prognosis for individuals with HFI is expected to be excellent [29]. However, some long-term complications have recently been described in patients on a FSS-restricted diet, such as hepatic steatosis [8]. In this study, we show that vitamin C deficiency is also common in HFI patients. Attention must be paid to detecting more potential sub-optimal outcomes in these patients to optimise their follow-up and treatment.

Strengths of this study include a comprehensive assessment of vitamin C and folate status, dietary intake and supplementation practices in a large nationwide cohort of HFI patients, compared to a cohort of age and sex- paired healthy control subjects.

We acknowledge that the statistical power of this study would increase with a larger number of participants. However, we were able to enrol a fairly good number of patients and healthy control subjects, considering the low prevalence of HFI. In addition, adherence to vitamin supplementation prescription in HFI patients was not assessed and, although the same researcher instructed all participants, the nutritional records of dietary intake could be subjective.

## Conclusions

Our results provide evidence for the first time supporting vitamin C supplementation in patients with HFI. There is great heterogeneity in vitamin supplementation practices and, despite follow-up at specialised centres, vitamin C insufficiency/deficiency is common in HFI patients. Further research is warranted to establish optimal doses of vitamin C and the need for folic acid supplementation in these patients.

## Data Availability

Additional data are available from the corresponding author on reasonable request.
