# Correction: Vitamin C and folate status in hereditary fructose intolerance

**DOI:** 10.1038/s41430-023-01334-3

**Published:** 2023-09-06

**Authors:** Ainara Cano, Carlos Alcalde, Amaya Belanger-Quintana, Elvira Cañedo-Villarroya, Leticia Ceberio, Silvia Chumillas-Calzada, Patricia Correcher, María Luz Couce, Dolores García-Arenas, Igor Gómez, Tomás Hernández, Elsa Izquierdo-García, Dámaris Martínez Chicano, Montserrat Morales, Consuelo Pedrón-Giner, Estrella Petrina Jáuregui, Luis Peña-Quintana, Paula Sánchez-Pintos, Juliana Serrano-Nieto, María Unceta Suarez, Isidro Vitoria Miñana, Javier de las Heras

**Affiliations:** 1 Biocruces Bizkaia Health Research Institute, 48093 Barakaldo, Spain; 2 Food Research, AZTI, Basque Research and Technology Alliance (BRTA), Parque Tecnológico de Bizkaia, Astondo Bidea, Edificio 609, 48160 Derio, Spain; 3 Paediatrics Unit, Río Hortega University Hospital, 47012 Valladolid, Spain; 4 Metabolic Diseases Unit, Department of Paediatrics, Ramón y Cajal Hospital, 28034 Madrid, Spain; 5 Department of Metabolism Diseases and Nutrition, Niño Jesús University Children´s Hospital, 28009 Madrid, Spain; 6 Internal Medicine Service, Cruces University Hospital, 48903 Barakaldo, Spain; 7 12 de Octubre University Hospital, CIBERER, MetabERN, 28041 Madrid, Spain; 8 Nutrition and Metabolic diseases Unit, La Fe University Hospital, 46026 Valencia, Spain; 9 Unit of Diagnosis and Treatment of Congenital Metabolic Diseases, Department of Pediatrics, IDIS-Health Research Institute of Santiago de Compostela. CIBERER. MetabERN. Santiago de Compostela University Clinical Hospital, 15704 Santiago de Compostela, Spain; 10 Department of Paediatric Gastroenterology, Hepatology and Nutrition, Sant Joan de Déu Hospital, 08950 Barcelona, Spain; 11 Araba University Hospital, 01009 Vitoria-Gasteiz, Spain; 12 Paediatric Service, Albacete University Hospital, 02006 Castilla-La Mancha, Spain; 13 Pharmacy Department, Infanta Leonor University Hospital, 28031 Madrid, Spain; 14 Clinical Nutrition Section, Navarra University Hospital, 31008 Pamplona, Spain; 15 Paediatric Gastroenterology, Hepatology and Nutrition Unit, Mother and Child Insular University Hospital complex, Asociación Canaria para la Investigación Pediátrica (ACIP), CIBEROBN. University Institute for Research in Biomedical and Health Sciences, University of Las Palmas de Gran Canaria, 35016 Las Palmas de Gran Canaria, Spain; 16 Paediatric Service, Málaga Regional University Hospital (HRU), 29010 Málaga, Spain; 17 Biochemistry Laboratory, Metabolism Area, Cruces University Hospital, 48903 Barakaldo, Spain; 18 Division of Paediatric Metabolism, CIBERER, MetabERN, Cruces University Hospital, 48093 Barakaldo, Spain; 19 Department of Paediatrics, University of the Basque Country (UPV/EHU), 48940 Leioa, Spain

**Keywords:** Metabolic disorders, Nutrition

Correction to: *European Journal of Clinical Nutrition* 10.1038/s41430-022-01178-3, published online 19 July 2022

In the original article [1], there was a mistake in the calculation of the *p* values in the comparisons of the categorical variables. These *p* values have been corrected in Table 1 and Fig. 2.
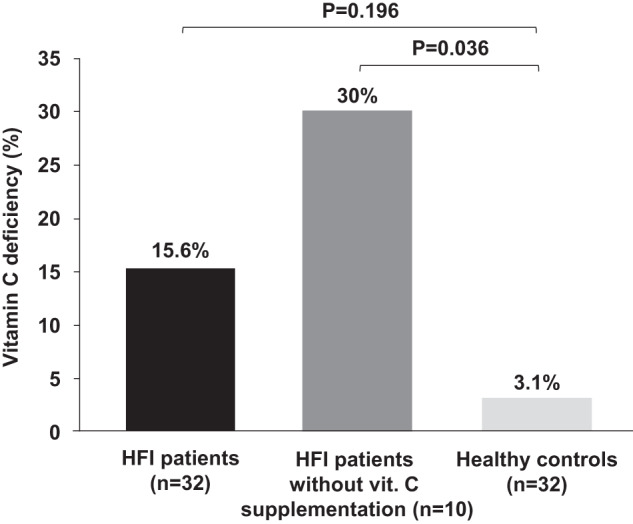

**Table 1:** Clinical, biochemical, and nutritional characteristics in HFI patients and healthy controls.

	Healthy controls	HFI patients	*p* value
*n*	32	32	
Male/female, *n*/*n*	13/19	12/20	0.798
Age, years	16.0 [2.1–61.3]	14.6 [5.5–63.5]	0.961
Weight, kg	50.5 ± 15.3	47.2 ± 15.6	0.401
BMI, kg/m^2^	20.2 ± 3.2	19.0 ± 3.1	0.710
Plasma vitamin C (µmol/L)	45.1 [10.2–129.5]	49.4 [5.7–138.0]	0.895
Serum folate (nmol/L)	21.5 [12.7–48.7]	24.7 [6.1–54.4]	0.619
Vitamin C hypovitaminosis/deficiency (*n*; %)	2; 6.3%	7; 21.9%	0.148
Vitamin C deficiency (*n*; %)	1; 3.1%	5; 15.6%	0.196
Folate deficiency (*n*; %)	0	1; 3.1%	1.000
**Dietary intake**			
*n*	28	30	
Vegetable fiber (g/day)	15.9 [9.4–59.7]	12.1 [6.1–21.4]	**0.006**
Vitamin C (mg/day)	107.0 [42.8–346.6]	23.8 [6.4–76.1]	**<0.001**
Folate (µg/day)	202.2 [115.8–524.2]	183.5 [78.6–304.3]	**0.027**

Continuous variables are represented as mean ± standard deviation or as median [minimum–maximum], depending on data distribution. Significant *p* values are marked in bold. Body mass index (BMI). Vitamin C hypovitaminosis/deficiency: circulating vitamin C levels ≤23 µmol/L. Vitamin C deficiency: circulating vitamin C levels ≤11 µmol/L.

In the abstract, the following sentence “Interestingly, a higher percentage of non-supplemented HFI patients were vitamin C deficient when compared to supplemented HFI patients (30% vs 9.1%; *p* = 0.01) and to healthy controls (30% vs 3.1%; *p* < 0.001)” should read as follows: “Interestingly, a higher percentage of non-supplemented HFI patients were vitamin C deficient when compared to healthy controls (30% vs 3.1%; *p* = 0.036).”

In the ‘Vitamin C status’ section, the following excerpt contained some mistakes: “Although 22 out of 32 HFI patients received vitamin C supplementation, there were no differences in plasma vitamin C levels between HFI patients and healthy controls (Table 1). However, there was a higher percentage of vitamin C deficient patients in the HFI group compared to the healthy controls (15.6% vs. 3.1%; *p* < 0.001) (Fig. 2). Taking into account vitamin C supplementation, although there were no significant differences in vitamin C levels between non-supplemented HFI patients and healthy controls (32.9 [5.7–76.7] mol/L vs. 45.1 [10.2–129.5] mol/L; *p* = 0.154), a higher percentage of non-supplemented HFI patients presented vitamin C deficiency (30% vs. 3.1%; *p* < 0.001) (Fig. 2).

Within the HFI group, the patients that were not given vitamin C supplements presented lower circulating levels than those who were given supplements (32.9 [5.7–76.7] mol/L vs. 59.1 [6.8–138] mol/L; *p* = 0.047) and a higher percentage of these non-supplemented patients displayed vitamin C deficiency (30% vs. 9.1%; *p* = 0.01). The amount of vitamin C supplementation and plasma levels correlated positively (*R* = 0.443; *p* = 0.011) (Fig. 3)”. It has been corrected as follows: ”Twenty-two out of 32 HFI patients received vitamin C supplementation, and there were no differences in plasma vitamin C levels or the percentage of vitamin C deficiency between HFI patients and healthy controls (Table 1). Taking into account vitamin C supplementation, although there were no significant differences in vitamin C levels between non-supplemented HFI patients and healthy controls (32.9 [5.7–76.7] mol/L vs. 45.1 [10.2–129.5] mol/L; *p* = 0.154), a higher percentage of non-supplemented HFI patients presented vitamin C deficiency (30% vs. 3.1%; *p* = 0.036) (Fig. 2).

Within the HFI group, although the patients that were not given vitamin C supplements presented lower circulating levels than those who were given supplements (32.9 [5.7–76.7] mol/L vs. 59.1 [6.8–138] mol/L; *p* = 0.047), there were not statistically significant differences in the percentage of vitamin C deficiency between the two groups (30% vs. 9.1%; *p* = 0.293). The amount of vitamin C supplementation and plasma levels correlated positively (R = 0.443; *p* = 0.011) (Fig. 3).”

In the section ‘Multivitamin vs. single supplementation’, the sentence “In addition, although there were no statistically significant differences in circulating vitamin C levels between HFI patients with single and multivitamin supplementation (67.0 [21.6–94.8] mol/L vs. 22.7 [6.8–103.3] mol/L; *p* = 0.368), a higher percentage of HFI patients on multivitamin supplementation presented vitamin C deficiency (25% vs. 0%; *p* < 0.001)” was corrected to read “There were no statistically significant differences in circulating vitamin C levels (67.0 [21.6–94.8] mol/L vs. 22.7 [6.8–103.3] mol/L; *p* = 0.368), or in the percentage of vitamin C deficiency (28.6% vs. 0%; *p* = 0.137) between HFI patients with single and multivitamin supplementation.”

Finally, in the ‘Discussion’ section, the sentence “The most relevant finding of the present study is that the HFI patients that did not consume vitamin C supplements presented a higher percentage of vitamin C deficiency than the healthy control subjects or HFI patients that received vitamin supplementation, providing for the first time evidence for the indication of vitamin C supplementation in patients with HFI under a FSS-restricted diet” was slightly adjusted to read “The most relevant finding of the present study is that the HFI patients that did not consume vitamin C supplements presented a higher percentage of vitamin C deficiency than the healthy control subjects, providing for the first time evidence for the indication of vitamin C supplementation in patients with HFI under a FSS-restricted diet.”

The authors apologize for these errors and state that these do not change the scientific conclusions of the article. The original article has been corrected.
